# Growth Regulated Oncogene-α Upregulates TNF-α and COX-2 and Activates NOD1/RIPK2 mediated-MAPK Pathway in Head and Neck Squamous Cell Carcinoma

**DOI:** 10.7150/jca.82300

**Published:** 2023-04-09

**Authors:** Leong-Perng Chan, Ya-Ping Tseng, Hui-Ching Wang, Chen-Yu Chien, Che-Wei Wu, Ling-Feng Wang, Chia-Hua Liang

**Affiliations:** 1 Department of Otorhinolaryngology-Head and Neck Surgery, Kaohsiung Medical University Hospital, Faculty of Medicine, College of Medicine, Kaohsiung Medical University, Kaohsiung, Taiwan; 2 Department of Otorhinolaryngology-Head and Neck Surgery, Kaohsiung Municipal Ta-Tung Hospital, Kaohsiung Medical University, Kaohsiung, Taiwan; 3 Institute of Basic Medical Sciences, National Cheng Kung University, Tainan, Taiwan; 4 Department of Internal Medicine, Division of Hematology and Oncology, Kaohsiung Medical University Hospital, Kaohsiung Medical University, Kaohsiung, Taiwan; 5 Department of Otolaryngology-Head and Neck Surgery, Kaohsiung Municipal Siaogang Hospital, Kaohsiung Medical University, Kaohsiung, Taiwan; 6 Department of Cosmetic Science and Institute of Cosmetic Science, Chia Nan University of Pharmacy and Science, Tainan, Taiwan

**Keywords:** HNSCC, Groα, NOD1, MAPK, Metastasis

## Abstract

**Purpose:** The long-term prognosis and survival rate of patients with recurrent or metastatic head and neck squamous cell carcinoma (HNSCC) are poor, although the identification of specific biomarkers that reveal its nature and aggressiveness has improved it. Growth-related oncogene alpha (Groα) and NOD1 (nucleotide-binding oligomerization domain 1) can be used as prognosis markers to identify subgroups of HNSCC patients with low survival rates and as potential therapeutic targets for HNSCC patients. However, the mechanism associated with the Groα-mediated NOD pathway in HNSCC progression remains unclear.

**Method:** Overall survival analysis and multiple-gene comparison were analyzed using Gene Expression Profiling Interactive Analysis (GEPIA). qRT-PCR and RT-PCR were used to analyze mRNA expression. Microarray, immunofluorescence staining or western blot analyses were carried out to detect protein expression.

**Results:** Groα was significantly higher in the grade 4 HNSCC tumor tissues compared with that in grade 1-3 and healthy subjects. High expression of Groα, NOD1 and RIPK2 (receptor-interacting serine-threonine kinase 2) is correlated with survival rate in HNSCC patients. Treatment of SCC25 and OECM-1 cells with Groα increased the expression of NOD1 and RIPK2 in a concentration-dependent manner. The findings herein reveal the association of Groα, NOD1 and RIPK2 biomarkers with HNSCC carcinogenesis. Moreover, Groα is the major stimulus of inflammatory mediation and promotes TNF-α (tumor necrosis factor-α) and COX-2 (cyclooxygenase-2) expression in HNSCC. Groα induces TNF-α and COX-2 expression through regulation involving ERK (extracellular signal-regulated kinase)-, JNK (C-Jun N-terminal kinase)- and p38 MAPK (mitogen-activated protein kinase)-dependent signaling pathways.

**Conclusions:** Our findings herein constitute the first evidence that Groα is important in HNSCC progression and metastasis via the NOD1-mediated MAPK pathway, suggesting a role for Groα and NOD1 in mediating metastasis and its potential as a therapeutic target.

## Introduction

Squamous cell carcinoma (SCC) is the most common malignant tumor of the head and neck (HN) and generally detected in the late stages when the cancer has advanced. HNSCC occurs in various locations, including the inner lip, oral cavity, floor of the mouth, larynx, and pharynx. Antibody drugs for treating HNSCC, such as nivolumab, cetuximab, and pembrolizumab, have been developed. However, the five-year survival rate of patients with HNSCC has remained at ~50% for the past 30 years, despite therapeutic advances 1. Therefore, the identification of more accurate prognosis biomarkers and suitable therapy targets remains the primary focus of HNSCC research.

Chemokines are small molecules that are secreted by cells that have an essential role in chemotaxis and angiogenesis and are moderators of tumor initiation and metastasis 2. Growth-related oncogene alpha (Groα), also known as CXCL1 (C-X-C motif chemokine ligand 1), is a member of the chemokine (chemotactic cytokines) family and is significantly up-regulated in various inflammatory diseases. It also has a critical role in the setting the tumor microenvironment, tumor progression, metastasis and angiogenesis 3. A previous report implied that Groα can be used as a prognosis marker to identify subgroups of HNSCC patients with low survival rates and it may be a suitable therapeutic target for such patients 4.

Toll-like receptors (TLRs) and Nod-like receptors (NLRs) are two major classes of innate immune sensor, which provide immediate responses against pathogenic invasion or tissue injury 5. As a fundamental member of NLR, the NOD1 (nucleotide-binding oligomerization domain 1) has an important role in the induction of innate immune and inflammatory responses. NOD1 has an important role not only in defending hosts against pathogens but also in maintaining tissue homeostasis by regulating cell apoptosis, and the inflammatory and tissue repair responses to injury. Recent studies have shown that NOD1 has an important role in controlling tumorigenesis 6,7. Several crucial proteins, NOD1, RIPK2 (receptor-interacting serine-threonine kinase 2), caspase-12 and human β Defensin 1, 2, 3 (hBD1, 2, 3) participate in or regulate the NOD1 signaling pathway 8. Our earlier study demonstrated that higher NOD1 and RIPK2 expressions were detected in the tissues of HNSCC patients than in NCMT (non-cancerous matched tissue), and identified a significant relationship between the increasing NOD1/2 expression and HNSCC progression 9. NOD1/caspase activation recruitment domain 4 (CARD4) and NOD2/CARD15 gene polymorphisms may be associated with an altered risk of developing various tumors 10. NOD1/2 interacts with the CARD-containing RIPK2, activating the nuclear factor kappa B (NF-κB) pathway and MAPK (mitogen-activated protein kinase) pathways 11. At least three MAPK families have been characterized; they are C-Jun *N*-terminal kinase/stress-activated protein kinase (JNK/SAPK), extracellular signal-regulated kinase (ERK), and p38 kinase 12. p38 MAPK and NF-κB regulate COX-2 (cyclooxygenase-2) expression in response to TNF-α (tumor necrosis factor-α), interleukin (IL)-1β, and interferon (IFN)-γ, which is composed of complex inflammatory stimuli 13. A recent study has demonstrated that Groα that is released by spinal astrocytes following inflammation acts on CXCR2 (CXC receptor 2)-expressing spinal neurons to increase ERK activation, synaptic transmission and COX-2 expression in dorsal horn neurons and contributes to the pathogenesis of inflammatory pain 14. Inhibition of the MAPK signaling cascade pathway resulted in a statistically significant reduction of Groα protein secretion 15. However, the relationships between 1) the NOD pathway and 2) the MAPK signaling pathway and the expression of Groα from HNSCC progression are poorly understood. This study establishes that Groα affects inflammatory mediators; regulates the NOD and MAPK pathways and is related to HNSCC progression.

## Materials and Methods

### Patient selection and specimens

Gene Expression Profiling Interactive Analysis (GEPIA) (http://gepia.cancer-pku.cn/) is a website that uses standard processing methods to analyze the mRNA expression data of tumor and normal samples from the Cancer Genome Atlas (TCGA) projects. The expression level, survival rate, and multiple gene comparison were conducted using GEPIA. We also used UALCAN (http://ualcan.path.uab.edu/), which is a website resource for analyzing cancer OMICS data.

This clinical research was approved by the ethics committee of the Kaohsiung Medical University Hospital (KMUHIRB-G(II)-20180051). Sixty-two adult patients were recruited for the trial between Feb. 2019 and Sep. 2021 from the Department of Otolaryngology-Head and Neck Surgery, Kaohsiung Medical University Hospital, Kaohsiung Medical University, Kaohsiung, Taiwan.

Table 1 summarizes the clinical characteristics of the patients and 90% for all HNSCC patients are squamous cell carcinoma. Patients with documented HNSCC of the oral cavity, oropharynx, larynx and hypopharynx were recruited. The ages of the patients ranged from 34 to 78 years (mean, 57.3 years). In total, 62 patients with HNSCC, including 58 males (93.5%) and 4 females (6.5%). The tumor locations were 55 in the oral cavity (88.7%), 1 in the oropharynx (16.1%), and 6 in the hypopharynx (4.8). 9 patients presented with well differentiation (14.5%), 47 patients with moderate (75.8%) and 6 patients with poor (9.7%). Half of the patients had late-stage (74.2%) and the others had early-stage (25.8%). Lymph node metastasis occurred in 32 patients (51.6%) and the remaining had 30 patients not lymph node metastasis (48.4%), were analyzed in this retrospective study. All patients had recently been diagnosed with a primary disease, and had not received any treatment in the form of chemotherapy, radiotherapy or alternative remedies.

To isolate RNA and protein, tissues, is from biopsies, were placed in fresh-frozen liquid nitrogen and underwent purification for further use in microarray, qRT-PCR, RT-PCR and western blot analyses. For immunofluorescence analysis, HNSCC tissues were fixed in buffered formalin and embedded in paraffin, sliced into 3 μm-thick sections, and mounted on glass slides. Table 2 summarizes relevant information concerning the HNSCC patients.

### Microarray analyses

NCMT (NCMT was sampled 5 cm away from the resection margin) and HNSCC tissue RNA were extracted using Trizol reagent (Invitrogen, Carlsbad, CA, USA), and then the RNeasy Mini Kit (Qiagen, Hilden, Germany) was used to quantify the isolated. RNA isolated at OD 260 nm and qualitatively analyzed using a bioanalyzer (Agilent Technology, USA). A 0.2 μg mass of total RNA was amplified using a Low-Input Quick-Amp Labeling Kit (Agilent Technologies, USA) and labeled with Cy3 (CyDye, Agilent Technologies, USA) during an *in vitro* transcription process. A 0.6 μg mass of Cy3-labled cRNA was fragmented to a mean size of approximately 50-100 nucleotides by incubation with a fragmentation buffer at 60°C for 30 minutes. Correspondingly fragmented labeled cRNA was then pooled and hybridized using an Agilent Sure Print G3 Human V2 GE 8 × 60 K Microarray (Agilent Technologies, USA) at 65°C for 17 h. After the microarrays were washed and dried by blowing with a nitrogen gun, the microarrays were scanned using an Agilent microarray scanner (Agilent Technologies, USA) at 535 nm for Cy3. Scanned images are analyzed using Feature Extraction 10.5.1.1 software (Agilent Technologies, USA) for image analysis and normalization to quantify the signal and background intensity for each feature (Welgene Biotech. Co., Taipei, Taiwan). Raw signal data was normalized by quantile normalization for differential expressed genes discovering.

### Immunofluorescence staining analyses

Serial tissue sections were cut from the paraffin-embedded formalin-fixed tumor tissue and subjected to immunofluorescence and hematoxylin and eosin (HE) histological staining. Briefly, tissue sections were deparaffinized, hydrated, and stained using antibodies against Groα (1:400; bs-0863R), NOD1 (1:20000; orb-77656), NOD2 (1:50, sc-56168) and RIPK2 (1:100, bs-3546R) (Bioss Antibodies, USA), respectively, and an IgG isotype antibody (Santa Cruz, CA) served as negative control; then corresponding secondary antibodies with FITC. The section was also stained with Hoechst 33342 (Promega) to study the nuclear morphology. The specific protein expressions and cell nuclei were investigated under a fluorescence microscope (Nikon, TE2000-U, Japan).

### Cell culture

HNSCC SCC25 cells were obtained from the American Type Culture Collection (Rockville, MD). OECM-1 (SCC180) human oral cavity SCC cells were kindly provided by Prof. Hamm-Ming Sheu (National Cheng Kung University Medical College, Tainan, Taiwan). HNSCC cells were maintained in Dulbecco's modified Eagle medium (DMEM)/F12; OECM-1 cells were cultured in DMEM medium (GIBCO, Grand Island, NY) and 10% fetal bovine serum (FBS) (Hazelton Product, Denver, PA, USA). All cells were incubated at 37°C in a humidified atmosphere of 5% CO_2_ in air.

### RNA extraction, quantitative real-time polymerase chain reaction (qRT-PCR) and reverse transcription-PCR (RT-PCR) analyses

NCMT and HNSCC tissues RNA were extracted using the Total RNA Miniprep Purification Kit (GeneMark). To extract RNA from cultured cells, SCC25 and OECM-1 cells (1 × 10^5^ cells/ml) were treated with Groα (0, 1 and 10 nM) for 72 h, and then the RNA was isolated using the Trizol kit (Invitrogen, Carlsbad, CA, USA) and reverse transcribed using the PrimeScript™ 1st strand cDNA Synthesis Kit (Takara, Japan). qRT-PCR and RT-PCR were performed according to previously described protocol 9. The following primer sequences were used as follows: Groα, 5'-ACGTGAAGTCCCCCGGAC-3' and 5'-GCCCATTCTTGAGTGTGGCT-3'; NOD1, 5′-GAGATTGGCTTCTCCCCTTC-3′ and 5′-CTGCCCAGGCTCTCGTTGCT-3′; NOD2, 5′-AGCCATTGTCAGGAGGCTC-3′, and 5′-CGTCTCTGCTCCATCATAGG-3′; RIPK2, 5′- CCATTGAGATTTCGCATCCT-3′ and 5′-ATGCGCCACTTTGATAAACC-3′; TNF-α, 5′-TTCCTCACTCACACCATCAGCC-3′ and 5′-TGCCCAGATTCAGCAAAGTCC-3′; COX2, 5′-TGAGCATCTACGGTTTGCTG-3′ and 5′-TGCTTGTCTGGAACAACTGC-3′; JNK, 5′-CTGAAGCAGAAGCTCCACCA-3′ and 5′-CACCTAAAGGAGAGGGCTG-3′; ERK1/2, 5′-GCTCACCCTTACCTGGAACA-3′ and 5′-GGACCAGATCCAAAAGGACA-3′; p38, 5′-ATGAAGCTCTCCAACACCCG-3′ and 5′-GCACCTAAAGGAGAGGGCTG-3′; and β-actin, 5′-TCACCCACACTGTGCCCATCTACGA-3′ and 5′-CAGCGGAACCGCTCATTGCCAATCG-3′. The amplified RT-PCR products were electrophoresed on a 2% agarose gel, visualized by ethidium bromide staining and photographed under ultraviolet light. The relative expressions were determined by densitometry using the Image J software program, and normalized relative to β-actin.

### Western blot analyses

To extract protein from HNSCC tissues, frozen tissues were homogenized in lysis buffer (50 mM HEPES, pH 7.5, 150 mM NaCl, 10% glycerol, 1% Triton X-100, 1mM EDTA, 1 mM EGTA, 50 mM NaF, 1 mM sodium orthovanadate, 30 mM *p*-nitrophenyl phosphate, 10 mM sodium pyrophosphate, 1 mM phenylmethylsulfonyl fluoride, 10 μg/ml aprotinin, and 10 μg/ml leupeptin). Supernatants were collected and analyzed.

To extract protein from cultured cells, SCC25 and OECM-1 cells (1 × 10^5^ cells/ml) were treated with Groα (0, 1 and 10 nM) for 72 h, and then washed with ice-cold PBS before immediately being lysed using lysis buffer.

Tissue extracts and cell lysates were prepared and processed for western blotting with antibodies against Groα (bs-0863R) (Bioss Antibodies, USA), NOD1 (orb77656), NOD2 (sc-56168), RIPK2 (bs-3546R), TNF-α (sc-33639), COX2 (sc-166475), p-JNK (sc-6254), p-ERK (sc-81492), p-p38 (sc-166182) and β-actin (sc-47778) (Santa Cruz, CA). Proteins were visualized using ECL reagent and their relative expressions were determined by densitometry using the Image J software program, and normalized relative to β-actin.

### Statistical analysis

All numerical data were taken as mean ± standard deviation (SD). Student's *t*-test and one-way ANOVA were used to analyze the assays. Differences were considered statistically significant at *p* ≤ 0.05. The data were analyzed and plotted using software (SigmaPlot Version 8.0 and SigmaStat Version 2.03, Chicago, IL).

## Results

### GROα induces NOD-mediated signaling in HNSCC metastasis

**Figure 1:**
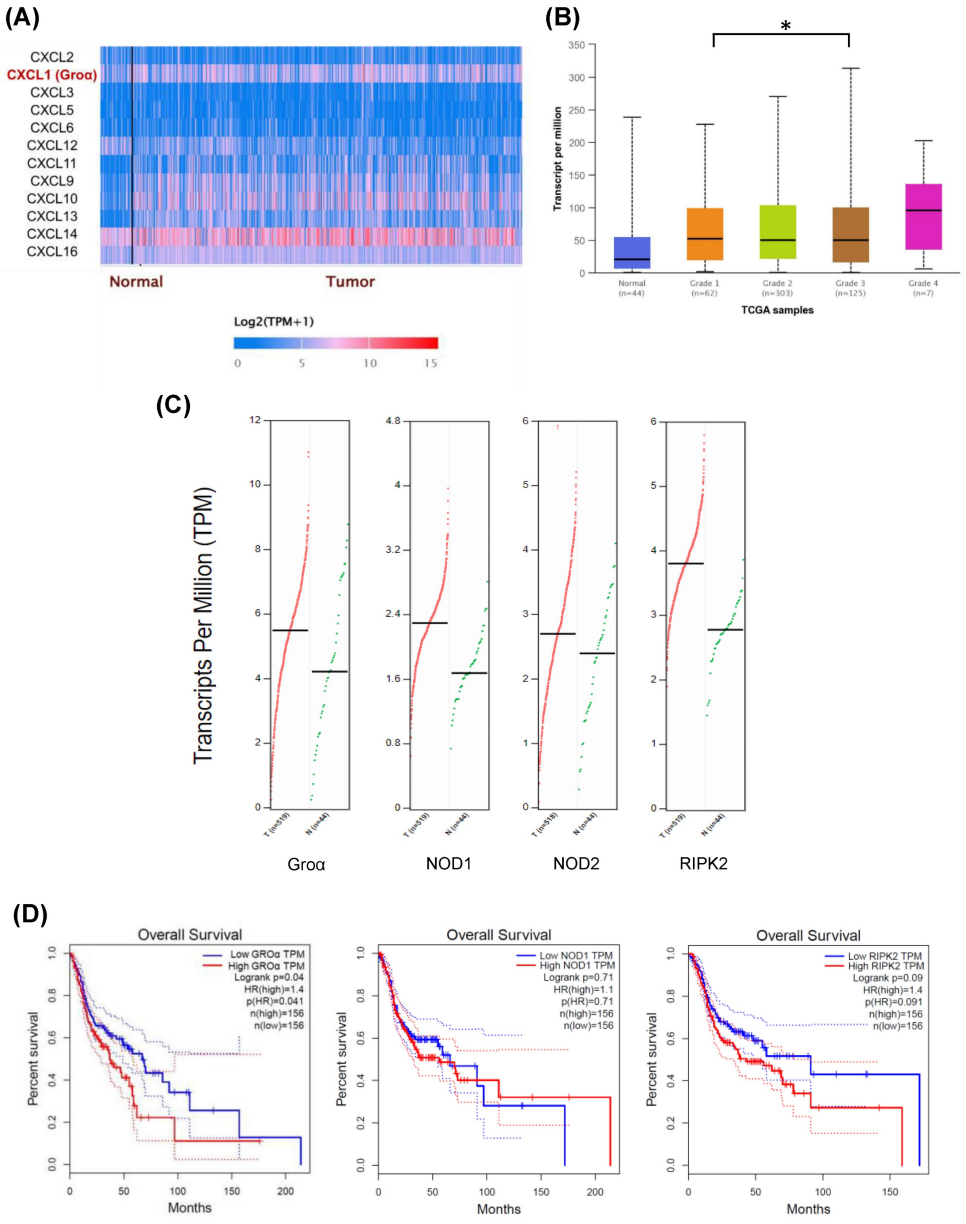
Expression levels of CXC family in HNSCC were assessed using UALCAN dataset (http://ualcan.path.uab.edu/index.html) **a** Difference in expressions between CXC family of HNSCC **b** Association of Groα mRNA expression with tumor grades in HNSCC patients. **c** The gene expression profile of Groα, NOD1, NOD2 and RIPK2 in HNSCC. **d** Overall survival analysis of Groα, NOD1, NOD2 and RIPK2 was performed using GEPIA tools.

In order to understand the factors of the CXC family of the inflammatory pathway in HNSCC, we searched the expression profile of the CXC family by using the UALCAN database. The expression of Groα and CXCL14 had higher expression in the HNSCC tumor (Fig. 1a). However, previous studies suggested CXCL14 expression had a negative association with all proinflammatory cytokines 16.

Besides, Groα was significantly higher in the grade 4 HNSCC tumor sample compared with that in grade 1-3 and normal samples (Fig. 1b). The abnormal expression of Groα may be associated with HNSCC progression. The online GEPIA tool analysis TCGA database indicates that the expressions of Groα, NOD1, NOD2 and RIPK2 in the cancer sample (*n*=519) are higher than those of in the normal tissue (*n*=44) (Fig. 1c). High Groα, NOD1 and RIPK2 levels also correlate with survival rate with HNSCC patients, especially Groα and RIPK2 (Fig. 1d). The patients were divided into early-stage and late-stage by number of lymph node metastasis and then we used the UALCAN database to analyze the statistical comparison of the Groα expression levels between the early- and late-stage. Analysis of HNSCC in the UALCAN database, the Groα expression between N0 and N3 (*p* < 0.02), was statistically significant.

**Figure 2:**
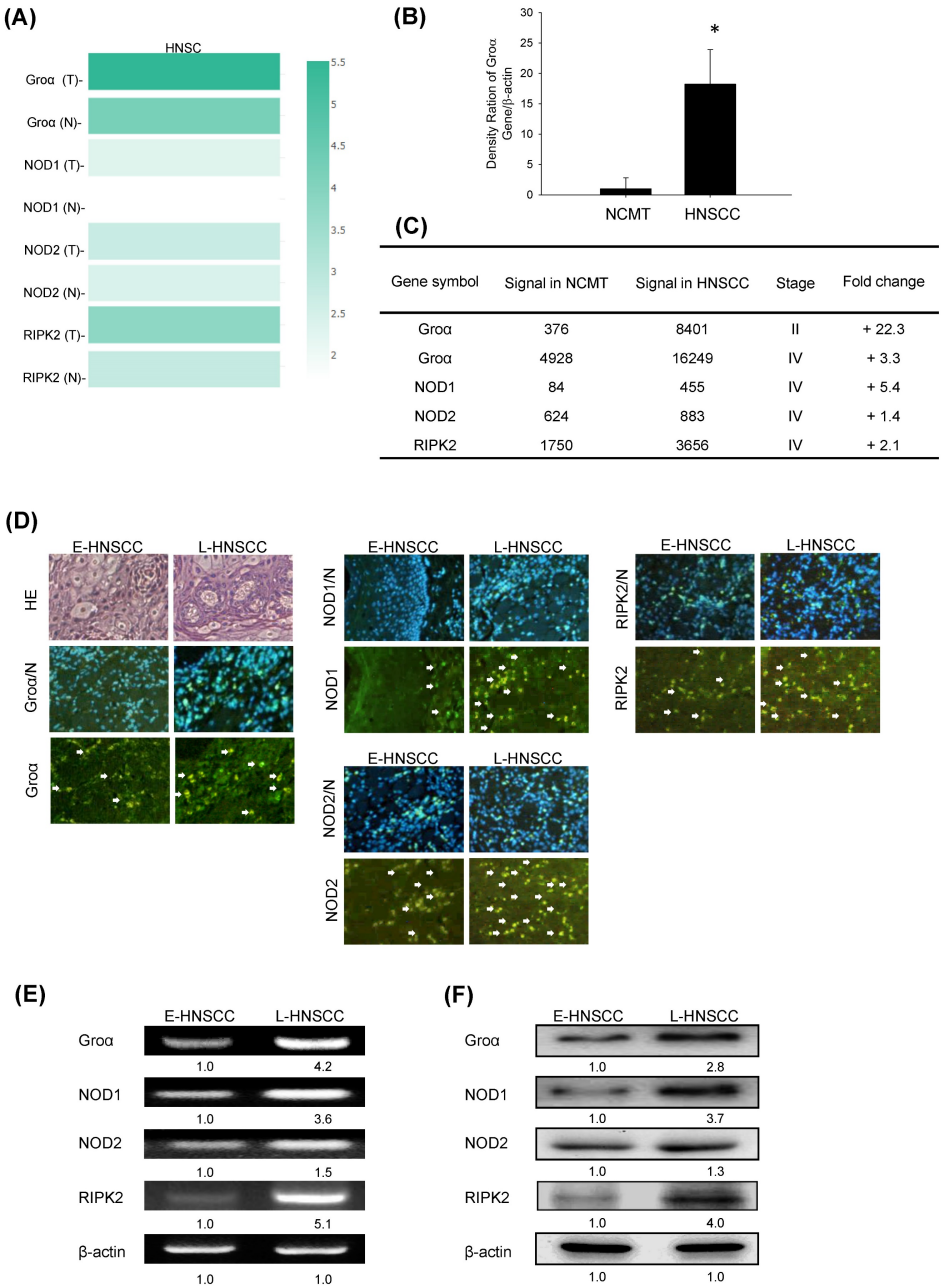
Groα and NOD-mediated signaling is strongly correlated with HNSCC progression. **a** Multiple-gene comparison was analyzed by using GEPIA, and the density of color represented the expression level of genes. **b** qRT-PCR was used to analyze Groα mRNA expression in 62 human HNSCC and NCMT tissues. Groα was more highly expressed in HNSCC than in NCMT.** c** Microarray analyses of the expression and fold-change thresholds of Groα, NOD1, NOD2 and RIPK2 in HNSCC and NCMT tissue (patients no. 1 and 2). NOD1 and RIPK2 expressions in advanced HNSCC were > 2 times higher than in NCMT, but NOD2 expression was only 1.4 times higher. **d** Groα, NOD1, NOD2 and RIPK2 expressions were higher in late-stage HNSCC (patient no. 4) than in early-stage HNSCC (patient no. 3), as confirmed by HE staining and immunofluorescence staining under an inverted fluorescent microscope (200× magnification). Levels of Groα, NOD1, NOD2 and RIPK2 in early-stage (E-) (patient no. 5) and late-stage (L-) (patient no. 6) HNSCC tissues were obtained using **e** RT-PCR and **f** western blot analysis. The expressions of Groα, NOD1 and RIPK2 in advanced HNSCC were significantly higher than in early-stage HNSCC, while the difference in the expression of NOD2 was not significant. Quantitative data reveal the L-HNSCC mRNA and protein level fold changes relative E-HNSCC, as determined by densitometry. β-actin was used as a loading control.

In addition, multiple-gene comparison analysis verified that the expression of these four genes in HNSCC samples was also higher than that in normal samples, indicating the potential of these genes in the diagnosis of HNSCC (Fig. 2a).

Groα expression in HNSCC was further analyzed. The mRNA expression levels of Groα in 62 human NCMT and HNSCC tissues were compared by qRT-PCR. Groα expression was significantly higher (18.2-fold) in HNSCC tissues than in NCMT (*p* < 0.05) (Fig. 2b). Microarray analysis was used to assess the mRNA expression of CXCL1 in NCMT and HNSCC tissues to verify the results of qRT-PCR analysis. As shown in Fig. 1b, the Groα level was higher (22.3-fold and 3.3-fold, respectively) in early-stage (cT2N0M0, patient no. 1) and late-stage (cT3N3bM0, patient no. 2) HNSCC than in NCMT tissues. These results suggest that Groα exhibited a cancer-specific response in HNSCC. Heatmap analysis showed the change of chemokines difference in early or the late stage of HNSCC. Groα was the highest of the CXC chemokine family in late stage and it was also a significantly upregulated expression between early or the late stage (Fig. S1).

Previous reports have found that NODs are functionally expressed in oral SCC cells and can trigger innate immune responses 9. The level of NOD1 in gastric tumor tissues is regulated above that in paired non-tumor samples 17. Our previous study demonstrated that NOD1 and RIPK2 expressions were higher in HNSCC tissue than NCMT, whereas NOD2 was weakly expressed in HNSCC tissue 9. In this investigation, an array analysis revealed that NOD1 and RIPK2 expressions in advanced HNSCC (cT3N3bM0, patient no. 2) were significantly up-regulated (> 2 times) above those in NCMT, being 5.4 and 2.1 times greater, respectively, but NOD2 was weakly expressed (1.4-fold) (Fig. 2c). Therefore, Groα, NOD1 and RIPK2 perform significantly better in early- or late-stage HNSCC than in NCMT.

The expression levels of Groα, NOD1, NOD2 and RIPK2 in early- and late-stage HNSCC tissues were evaluated using immunofluorescence staining analysis (Fig. 2d). The expression levels in late-stage HNSCC (patient no. 4) exceeded those in early-stage HNSCC (patient no. 3). The cytoplasm localization of Groα, NOD1, NOD2 and RIPK2 was associated with more consistent and stronger expression in advanced HNSCC than in early-stage HNSCC. RT-PCR and western blot analysis confirmed that the expressions of genes and proteins of Groα and the NOD1/2-mediated RIPK2 pathway were higher in late-stage (patient no. 6) than in early-stage (patient no. 5) HNSCC tissues (Figs. 2e and 2f). The expressions of Groα, NOD1 and RIPK2 in advanced HNSCC significantly exceeded those in early-stage HNSCC, while the difference in expressions of NOD2 was not as great significant. The experimental results confirmed that HNSCC was associated with consistent and strong Groα and NOD-mediated signaling expression.

**Figure 3:**
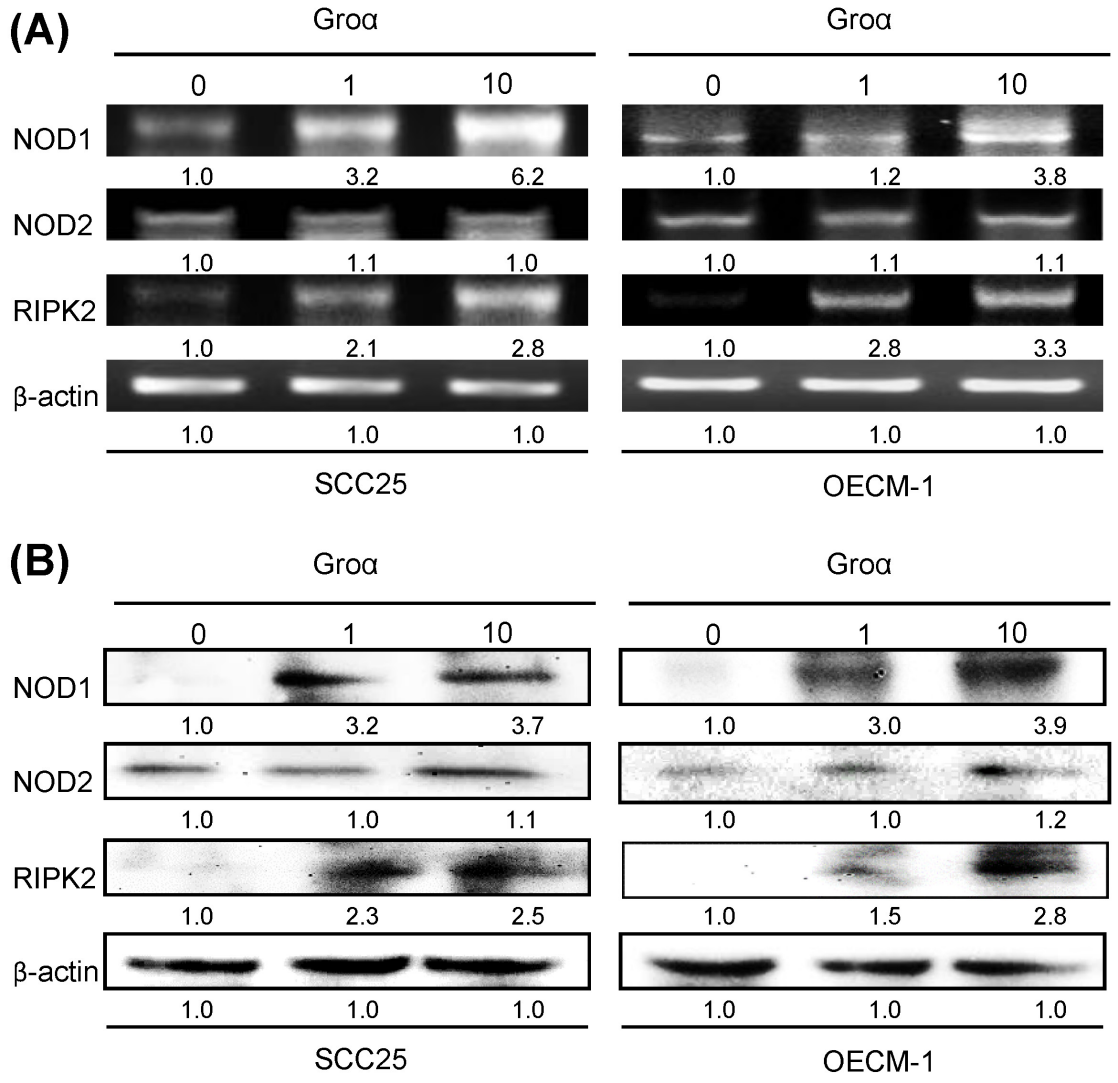
Groα promotes NOD signaling expression in HNSCC. SCC25 and OECM-1 cells were stimulated using Groα (0, 1 and 10 nM) for 72 h and levels of NOD1, NOD2 and RIPK2 were obtained using **a** RT-PCR and **b** western blot analysis. Exposure of SCC25 and OECM-1 cells to Groα increased the expressions of NOD1 and RIPK2 in a concentration-dependent manner, but the expression of NOD2 in the cells was unaffected by Groα treatment or was weak. Data reveal mRNA and protein fold-changes relative to untreated controls. β-actin was used as a loading control.

To define the NOD-mediated signaling pathway that is associated with Groα treatment, the gene and protein expressions of NOD1, NOD2 and RIPK2 in two types of HNSCC cells that had been treated with Groα for 72 h were obtained by RT-PCR and western blot analysis. As indicated in Figs. 3a and 3b, the exposure of SCC25 and OECM-1 cells to Groα (0, 1 and 10 nM) increased the expression of NOD1 and RIPK2 in a concentration-dependent manner. However, the expression of NOD2 in both types of HNSCC cells was unaffected by Groα treatment or weak as a result thereof. This finding suggests that the induction of HNSCC cancer progression by Groα is partially caused by the activation of NOD1 signaling partner RIPK2.

### GROα upregulates expressions of inflammatory mediator's TNF-α and COX-2 in HNSCC

**Figure 4:**
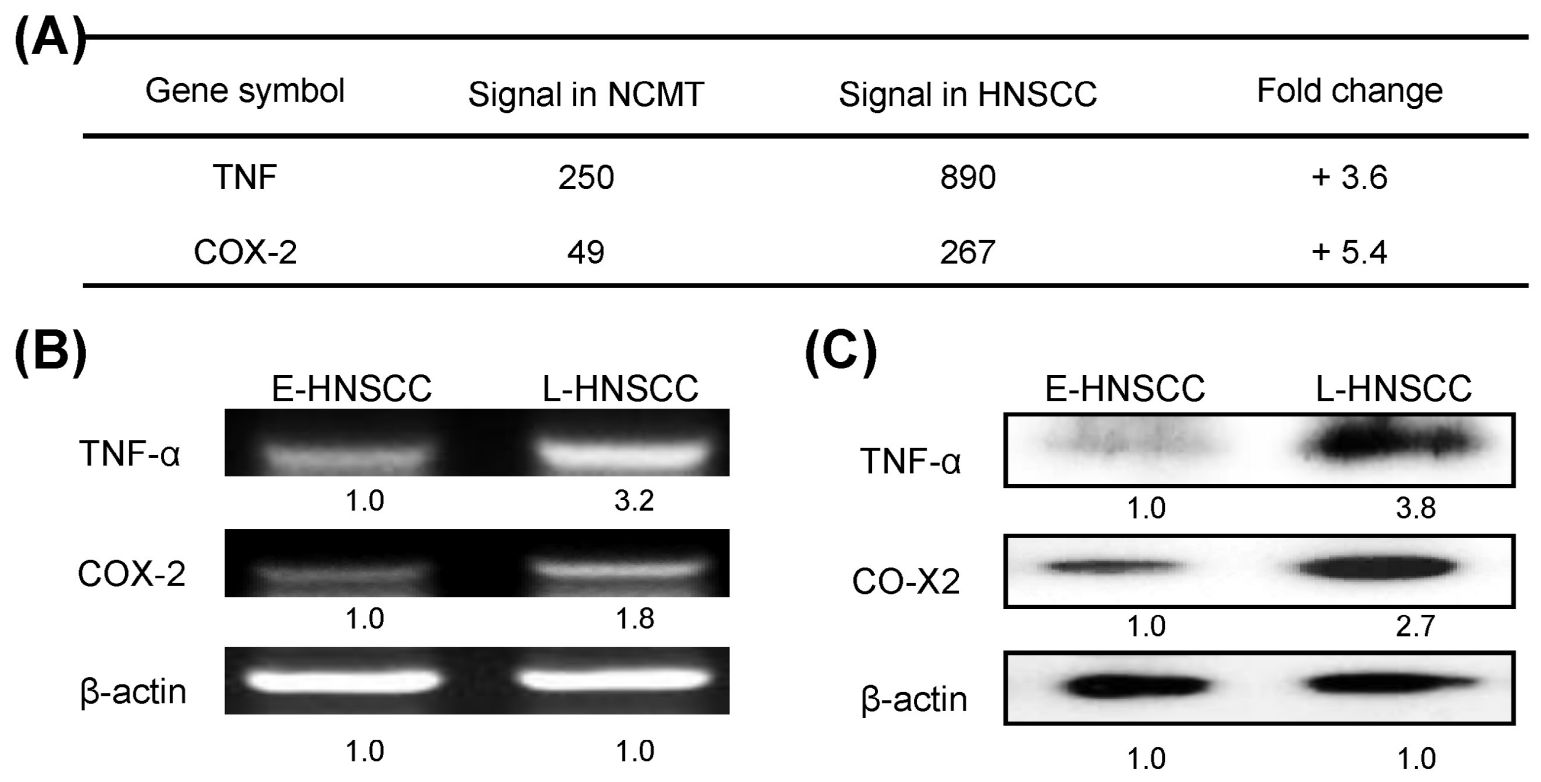
Expressions of inflammatory mediators TNF-α and COX-2 in advanced HNSCC. **a** Microarray analysis tests were used to evaluate the expression and fold-change thresholds of TNF-α and COX-2 in HNSCC and NCMT tissue (patient no. 2). TNF-α and COX-2 expressions in advanced HNSCC were > 2 times higher than in NCMT. Levels of TNF-α and COX-2 in early-stage (E-) (patient no. 7) and late-stage (L-) (patient no. 8) HNSCC tissues were obtained using **b** RT-PCR and** c** western blot analysis. Expressions of TNF-α and COX-2 in advanced HNSCC were significantly higher than in early-stage HNSCC. Quantitative data display L-HNSCC mRNA and protein fold changes relative E-HNSCC, as determined by densitometry. β-actin was used as a loading control.

A recent study showed that the immediate response of NOD activation might be caused by the production of TNF-α through NF-κB, TLR, and/or NOD signaling 18. In the present study, microarray technology demonstrated that the expressions of TNF and COX-2 genes in advanced HNSCC (patient no. 2) were significantly higher (3.6 and 5.4-fold) than in NCMT (Fig. 4a). RT-PCR and western blot analysis confirmed that the expressions of genes and proteins of TNF-α and COX-2 were higher in late-stage (patient no. 8) than in early-stage HNSCC tissues (patient no. 7) (Figs. 4b and 4c). TNF-α and COX-2 levels were higher (3.2 and 1.8-fold by RT-PCR, respectively; 3.8-, and 2.7-fold by western blot, respectively) in late-stage than in early-stage HNSCC tissues.

**Figure 5:**
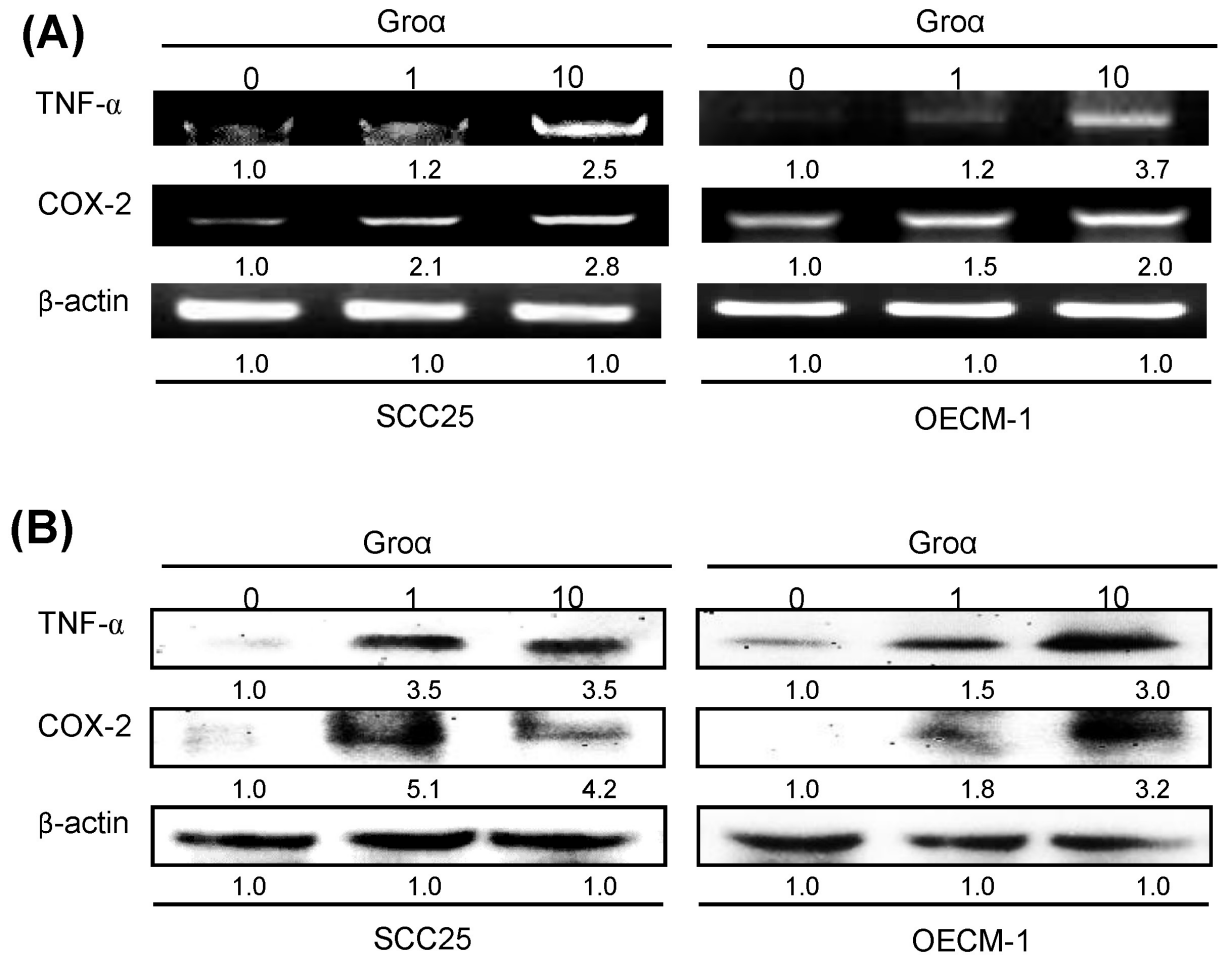
Groα stimulates expression of inflammatory mediators TNF-α and COX-2 in HNSCC. Upon exposure of SCC25 and OECM-1 cells to Groα (0, 1 and 10 nM) for 72 h, levels of TNF-α and COX-2 were obtained using **a** RT-PCR and **b** western blot analysis. Treatment with Groα significantly increased TNF-α and COX-2 expressions in SCC25 and OECM-1 cells. Data reveal mRNA and protein fold-changes relative to untreated controls. β-actin was used as a loading control.

To identify the inflammatory mediator that is associated with Groα treatment in HNSCC, the expressions of TNF-α and COX-2 in SCC25 and OECM-1 cells that were treated with Groα (0, 1 and 10 nM) for 72 h were determined by RT-PCR and western blot analysis. Treatment with Groα (10 nM) increased the TNF-α and COX-2 levels 2.5-fold and 2.8-fold respectively in SCC25 cells, and 3.7-fold and 2.0-fold respectively, in OECM-1, according to RT-PCR (Fig. 5a); and 3.5-fold and 4.2-fold respectively in SCC25 cells, and 3.0-fold and 3.2-fold respectively in OECM-1, according to western blot (Fig. 5b). These results suggest that Groα regulates inflammatory response by modulating TNF-α and COX-2 expressions in HNSCC.

### GROα upregulates MAPK signaling pathway in HNSCC

**Figure 6:**
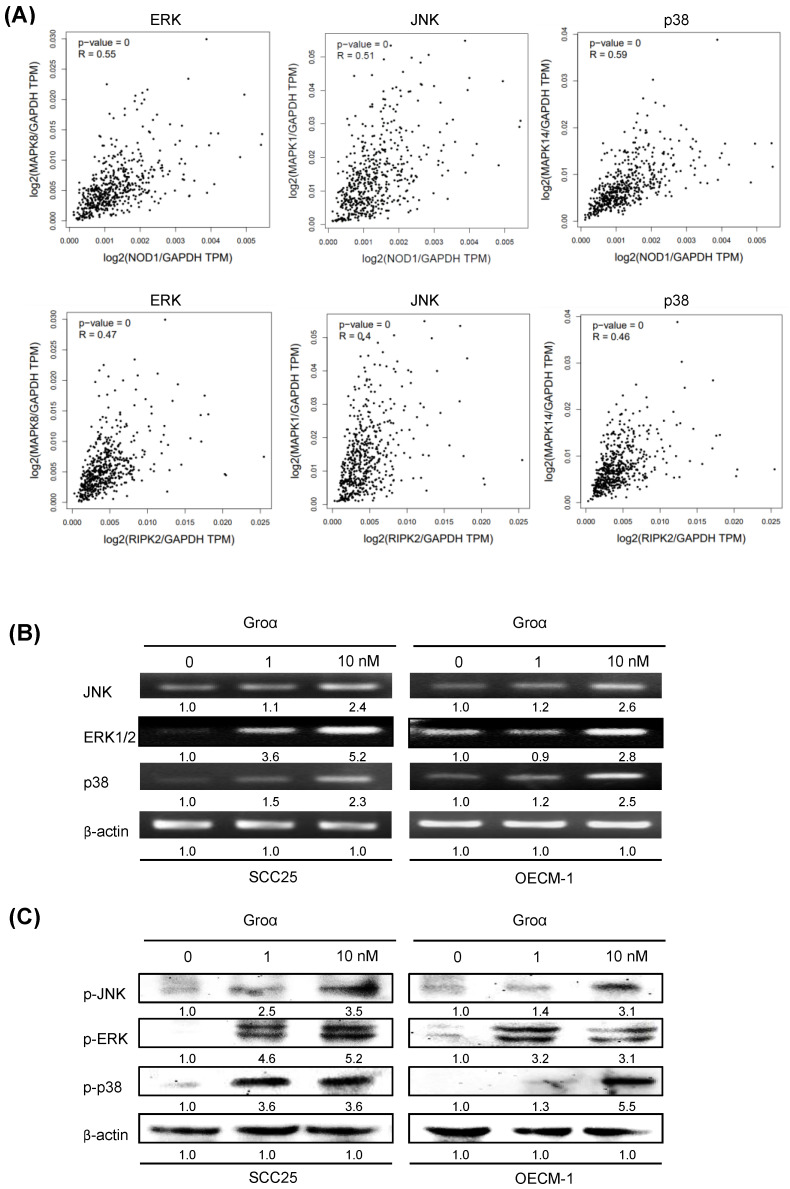
Groα stimulates expression of inflammatory mediators and upregulates MAPK signaling pathway in HNSCC. **a** Correlation analysis between NOD pathway (NOD1 and RIPK2) and inflammatory mediators (ERK, JNK, p38) in HNSCC samples from the GEPIA tool. Upon exposure of SCC25 and OECM1 cells to Groα (0, 0.1, 1 and 10 nM) for 72 h, levels of JNK, ERK and p38 MAPK were obtained using **b** RT-PCR and **c** western blot analysis. Exposure of SCC25 and OECM-1 cells to Groα increased the expressions of JNK, ERK and p38 MAPK in a concentration-dependent manner. Data reveal mRNA and protein fold-changes relative to untreated controls. β-actin was used as a loading control.

By analyzing the correlated genes in the HNSCC, the NOD1 and RIPK2 mRNA levels showed a positive correlation between ERK, JNK and p38 mRNA in the TCGA dataset (Fig. 6a). A previous report established that TNF-α induces Groα expression through the JNK, p38 MAPK and PI-3K/Akt signaling pathways in human pulmonary epithelial cells 15. To elucidate the association between Groα and the MAPK-mediated signaling pathway in HNSCC progression, the levels of JNK, ERK and p38, which are regulated in response to Groα, were determined in HNSCC cells. Upon exposure of SCC25 and OECM-1 cells to Groα (0, 1 and 10 nM) for 72 h, the expressions of JNK, ERK and p38 were determined by western blot and RT-PCR. Treatment with Groα increased JNK, ERK and p38 expressions in SCC25 cells and OECM-1 cells in a dose-dependent manner (Figs. 6b and 6c). Treatment with Groα (10 nM) increased JNK, ERK1/2 and p38 mRNA levels 2.4-, 5.2- and 2.3-fold respectively in SCC25 cells, and 2.6-, 2.8- and 2.5-fold respectively in OECM-1 cells (Fig. 6b). Western blot analysis was carried out to determine the expression of proteins p-JNK, p-ERK and p-p38 in Groα-treated HNSCC cells to verify the results of RT-PCR. Treatment with Groα (10 nM) increased the p-JNK, p-ERK and p-p38 levels 3.5-, 5.2- and 3.6-fold respectively in SCC25 cells, and 3.1-, 3.1- and 5.5-fold respectively in OECM1, according to western blotting (Fig. 6c). These results indicate that Groα induces TNF-α and COX-2 expression through regulation involving JNK-, ERK- and p38 MAPK-dependent signaling pathways.

## Discussion

Our previous cytokine array analysis study demonstrated that the expressions of IL-1β, IL-6, IL-8 and TNF-α in HNSCC (stage II) were significantly up-regulated > 2 times those in NCMT, and the up-regulation of IL-8 was the most significant. The expression of IL-8 was increased by a factor of 9.9 19. The profiles of secreted proteins in early- and late-stage HNSCC were compared herein to those in NCMT tissues. The Groα levels in the early-stage and late-stage HNSCC were higher (22.3-fold and 3.3-fold) than that in NCMT tissues. Levels were significantly higher in patients with HNSCC than in healthy subjects, suggesting that Groα was the most significantly differentially expressed chemokine.

NOD1 has an important role not only in defending hosts against pathogens but also in maintaining tissue homeostasis by regulating cell apoptosis, and the inflammatory and tissue repair responses to injury. The dysregulation of NOD1 signaling can cause persistent infection and chronic inflammation due to insufficient pathogen clearance, while chronic inflammation can initiate and promote cancer development 20. Disrupting the function of NOD1 makes MCF-7 breast cancer cells resistant to apoptotic cell death and promotes tumor growth in the body 21. NOD1 is thought to recruit the downstream effector RIPK2, which is not only a crucial in NOD1 signaling but also an important regulator of cellular proliferation, apoptosis and differentiation. A recent study found an association between RIPK2 polymorphism and the risk of urothelial cancer 22. Activation of the effector kinase RIPK2 induces the NF-κB and the MAPK pathways, activating inflammatory genes and producing antimicrobial polypeptides, such as hBD1, 2, and 3 8. Therefore, RIPK2 plays an important role in mediating inflammation and innate immunity.

Our earlier investigation identified higher NOD1 and RIPK2 expressions in HNSCC tissue than in NCMT, but only weak NOD2 expression 9. Human pharyngeal SCC (Detroit-562 and FaDu cells) exhibited strong expressions of NOD1 and NAIP but weak expressions of NOD2 and NLRP1 23, 24. FaDu cells displayed a lower Nod2 level and a higher NAIP level than healthy cells. In this study, Groα, NOD1, NOD2, and RIPK2 were significantly more strongly expressed in advanced HNSCC than in NCMT. Besides, the expressions of Groα, NOD1 and RIPK2 in advanced HNSCC were significantly higher than in early-stage HNSCC, while the difference in the performance of NOD2 was not significant. Groα, NOD1 and RIPK2 can potentially be used novel biomarkers for HNSCC carcinogenesis.

A recent study has shown that canonical members of the inflammatory senescence-associated secretory phenotype (SASP), such as IL1A, IL8, and Groα, are COX2-dependent. COX-2 is induced by pro-inflammatory cytokines at the site of inflammation and it promotes the COX-2-induced synthesis of prostaglandins; it stimulates cancer cell proliferation, promotes angiogenesis, and increases metastatic potential 25, 26. Additionally, the incubation of a primary culture of astrocytes with TNF-α markedly increased the levels of several chemokines, including CCL2 and CXCL1 27. TNF-α induces Groα expression in human A549 cells through transcriptional and nontranscriptional regulation that involves JNK-, p38 MAPK- and PI-3K/Akt-dependent signaling pathways 28. These results demonstrate that Groα induces TNF-α and COX-2 expression through regulation that involves MAPK-dependent signaling pathways.

## Conclusion

**Figure 7:**
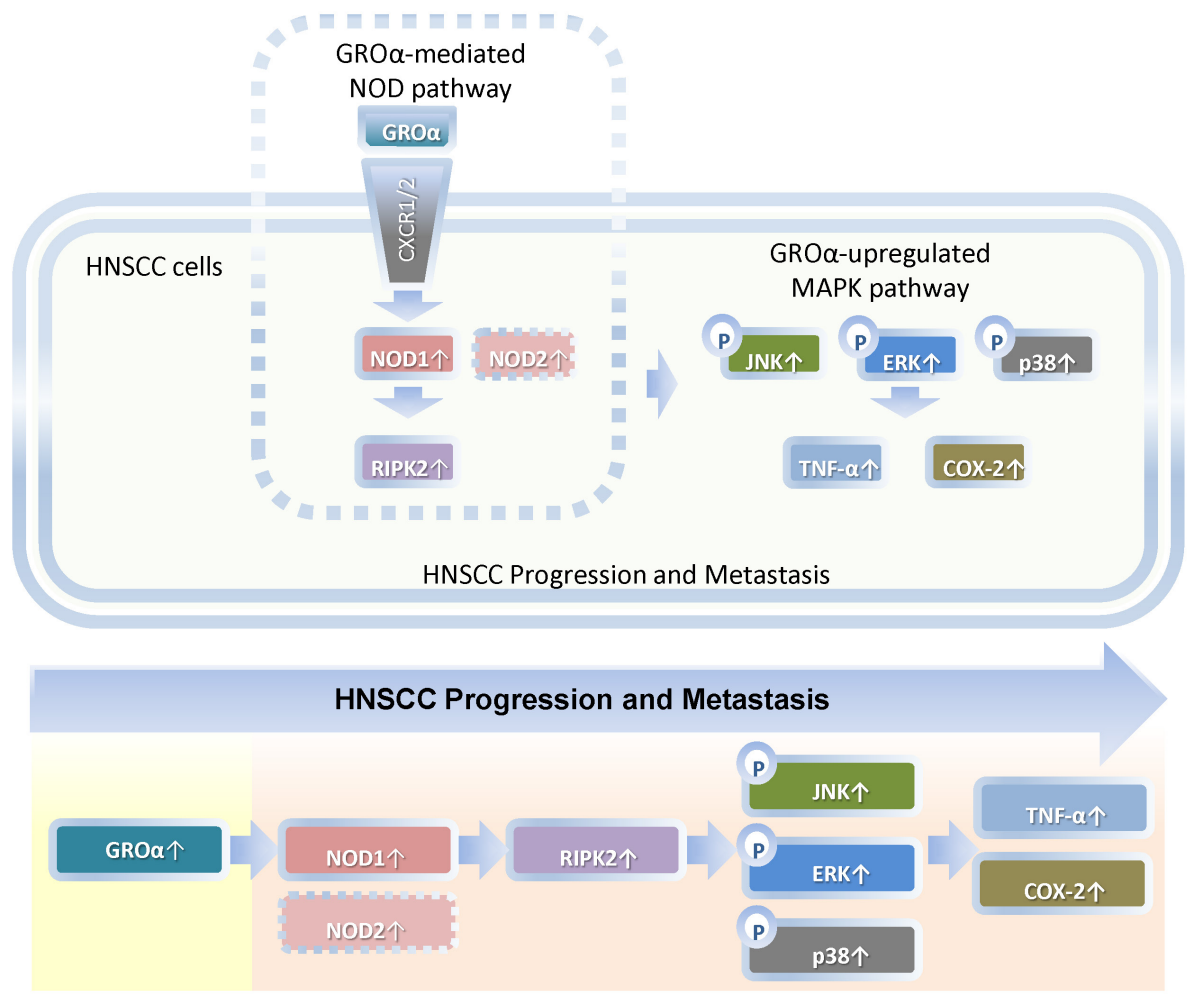
Schematic diagram by Groα promotes of expressions of inflammatory mediator TNF-α and COX-2 by activating NOD1/RIPK2-mediated JNK, ERK and p38 MAPK pathways in HNSCC progression and metastasis. TNF-α, tumor necrosis factor-α; COX-2, cyclooxygenase-2; nucleotide-oligomerization domain-containing protein 1, Groα; Growth regulated oncogene-α; NOD1; receptor-interacting protein kinase 2, RIPK2; C-Jun *N*-terminal kinase, JNK; extracellular signal-regulated kinase, ERK; p38 mitogen-activated protein kinase (p38 MAPK); HNSCC, head and neck squamous cell carcinoma.

Our findings constitute the first evidence that Groα is the major stimulus of inflammatory mediation and promotes TNF-α and COX-2 expression by activating NOD1/RIPK2-mediated JNK, ERK and p38 MAPK pathway in HNSCC progression and metastasis (Fig. 7). Future studies of the effects of Groα and NOD1-mediated MAPK signaling pathways that are associated with inflammation will establish their effectiveness as HNSCC biomarkers.

## Supplementary Material

Supplementary figure.

## Figures and Tables

**Table 1:** Clinical characteristics of HNSCC patients

Characteristics	Frequency	%
Numbers (n)	62	
Age (year)		
Mean	56.5	
Range	34-81	
Gender		
Male	58	93.5
Female	4	6.5
Location		
Oral cavity	55	88.7
Oropharynx	1	16.1
Hypopharynx	6	4.8
Differentiation grade		
Well	9	14.5
Moderate	47	75.8
Poor	6	9.7
Stage		
Early-stages (cT1-2N0M0)	16	25.8
Late-stages (cT1-4N1-3M0)	46	74.2
Lymph node metastasis		
N0	30	48.4
N1	13	21.0
N2	8	12.9
N3	11	17.7

**Table 2:** Summary of HNSCC patients

No.	Sex	Age (y)	Local	Stage
1	M	55	Oral cavity	cT2N0M0 Stage II
2	M	51	Oral cavity	cT3N3bM0 Stage IV
3	M	48	Oral cavity	cT1N0M0 Stage I
4	M	60	Hypopharynx	cT4aN0M0 Stage IV
5	F	52	Oral cavity	cT2N0M0 Stage II
6	M	57	Larynx	cT4aN0M0 stage IV
7	M	48	Oral cavity	cT2N0M0 Stage II
8	F	52	Oral cavity	cT4aN0M0 Stage IV

## Abbreviations

CARD4: caspase activation recruitment domain 4
COX-2: cyclooxygenase-2
CXCL1: C-X-C motif chemokine ligand 1
CXCR2: CXC receptor 2
ERK: extracellular signal-regulated kinase
GEPIA: Gene Expression Profiling Interactive Analysis
Groα: growth-related oncogene alpha
hBD: human β Defensin
HE: hematoxylin and eosin
HNSCC: head and neck squamous cell carcinoma
IFN: interferon
IL: interleukin
JNK: C-Jun *N*-terminal kinase
MAPK: mitogen-activated protein kinase
NCMT: non-cancerous matched tissue
NF-κB: nuclear factor kappa B
NLRs: Nod-like receptors
NOD1: nucleotide-binding oligomerization domain 1
RIPK2: receptor-interacting serine-threonine kinase 2
SAPK: stress-activated protein kinase
SASP: senescence-associated secretory phenotype
TCGA: The Cancer Genome Atlas
TLRs: Toll-like receptors
TNF-α: tumor necrosis factor-α

## References

[1] Cohen EEW, Bell RB, Bifulco CB, Burtness B, Gillison ML, Harrington KJ (2019). The society for immunotherapy of cancer consensus statement on immunotherapy for the treatment of squamous cell carcinoma of the head and neck (HNSCC). J Immunother Cancer.

[2] Asokan S, Bandapalli OR (2021). CXCL8 signaling in the tumor microenvironment. Adv Exp Med Biol.

[3] Wei LY, Lee JJ, Yeh CY, Yang CJ, Kok SH, Ko JY (2019). Reciprocal activation of cancer-associated fibroblasts and oral squamous carcinoma cells through CXCL1. Oral Oncol.

[4] Li Y, Wu T, Gong S, Zhou H, Yu L, Liang M (2021). Analysis of the prognosis and therapeutic value of the CXC chemokine family in head and neck squamous cell carcinoma. Front Oncol.

[5] Li P, Chang M (2021). Roles of PRR-mediated signaling pathways in the regulation of oxidative stress and inflammatory diseases. Int J Mol Sci.

[6] Topal Y, Gyrd-Hansen M (2021). RIPK2 NODs to XIAP and IBD. Semin Cell Dev Biol.

[7] Fernández-García V, González-Ramos S, Martín-Sanz P, Laparra JM, Boscá L (2021). NOD1-targeted immunonutrition approaches: on the way from disease to health. Biomedicines.

[8] Wang X, Jiang W, Duan N, Qian Y, Zhou Q, Ye P (2014). NOD1, RIP2 and Caspase12 are potentially novel biomarkers for oral squamous cell carcinoma development and progression. Int J Clin Exp Pathol.

[9] Chan LP, Wang LF, Chiang FY, Lee KW, Kuo PL, Liang CH (2016). IL-8 promotes HNSCC progression on CXCR1/2-mediated NOD1/RIP2 signaling pathway. Oncotarget.

[10] Ozbayer C, Kurt H, Bayramoglu A, Gunes HV, Metintas M, Degirmenci İ (2015). The role of NOD1/CARD4 and NOD2/CARD15 genetic variations in lung cancer risk. Inflamm Res.

[11] Yu H, Lin L, Zhang Z, Zhang H, Hu H (2020). Targeting NF-kappaB pathway for the therapy of diseases: Mechanism and clinical study. Signal Transduct Target Ther.

[12] Fabian DK, Fuentealba M, Dönertaş HM, Partridge L, Thornton JM (2021). Functional conservation in genes and pathways linking ageing and immunity. Immun Ageing.

[13] Das UN (2019). Bioactive lipids in intervertebral disc degeneration and its therapeutic implications. Biosci Rep.

[14] Cao DL, Zhang ZJ, Xie RG, Jiang BC, Ji RR, Gao YJ (2014). Chemokine CXCL1 enhances inflammatory pain and increases NMDA receptor activity and COX-2 expression in spinal cord neurons via activation of CXCR2. Exp Neurol.

[15] Lo HM, Lai TH, Li CH, Wu WB (2014). TNF-α induces CXCL1 chemokine expression and release in human vascular endothelial cells *in vitro* via two distinct signaling pathways. Acta Pharmacol Sin.

[16] Lv J, Wu ZL, Gan Z, Gui P, Yao SL (2020). CXCL14 overexpression attenuates sepsis-associated acute kidney injury by inhibiting proinflammatory cytokine production. Mediators Inflamm.

[17] Rommereim LM, Akhade AS, Dutta B, Hutcheon C, Lounsbury NW, Rostomily CC (2020). A small sustained increase in NOD1 abundance promotes ligand-independent inflammatory and oncogene transcriptional responses. Sci Signal.

[18] Ding W, Chim SSC, Wang CC, Lau CSL, Leung TY (2021). Molecular mechanism and pathways of normal human parturition in different gestational tissues: A systematic review of transcriptome studies. Front Physiol.

[19] Chan LP, Liu C, Chiang FY, Wang LF, Lee KW, Chen WT (2017). IL-8 promotes inflammatory mediators and stimulates activation of p38 MAPK/ERK-NF-κB pathway and reduction of JNK in HNSCC. Oncotarget.

[20] Philpott DJ, Sorbara MT, Robertson SJ, Croitoru K, Girardin SE (2014). NOD proteins: regulators of inflammation in health and disease. Nat Rev Immunol.

[21] Heim VJ, Stafford CA, Nachbur U (2019). NOD signaling and cell death. Front Cell Dev Biol.

[22] Jaafar RF, Ibrahim Z, Ataya K, Hassanieh J, Ard N, Faraj W Receptor-interacting serine/threonine-protein kinase-2 as a potential prognostic factor in colorectal Cancer Medicina (Kaunas). 2021;57:709.

[23] Millrud CR, Kvarnhammar AM, Tajti J, Munck-Wikland E, Uddman R, Cardell LO (2013). Nod-like receptors in head and neck squamous cell carcinoma. Acta Otolaryngol.

[24] Mey L, Jung M, Roos F, Blaheta R, Hegele A, Kinscherf R (2019). NOD1 and NOD2 of the innate immune system is differently expressed in human clear cell renal cell carcinoma, corresponding healthy renal tissue, its vasculature and primary isolated renal tubular epithelial cells. J Cancer Res Clin Oncol.

[25] Gonçalves S, Yin K, Ito Y, Chan A, Olan I, Gough S (2021). COX2 regulates senescence secretome composition and senescence surveillance through PGE2. Cell Rep.

[26] Desai SJ, Prickril B, Rasooly A (2018). Mechanisms of phytonutrient modulation of cyclooxygenase-2 (COX-2) and inflammation related to cancer. Nutr Cancer.

[27] Xu J, Zhu MD, Zhang X, Tian H, Zhang JH, Wu XB (2014). NFkappaB-mediated CXCL1 production in spinal cord astrocytes contributes to the maintenance of bone cancer pain in mice. J Neuroinflammation.

[28] Shieh JM, Tsai YJ, Tsou CJ, Wu WB (2014). CXCL1 regulation in human pulmonary epithelial cells by tumor necrosis factor. Cell Physiol Biochem.

