# Supplementary material for: Growth Regulated Oncogene-α Upregulates TNF-α and COX-2 and Activates NOD1/RIPK2 mediated-MAPK Pathway in Head and Neck Squamous Cell Carcinoma
Source: J Cancer. 2023 Apr 9;14(6):989–1000. doi: 10.7150/jca.82300 (PMC10158519; doi:10.7150/jca.82300)
Supplement: Supplementary file 1 — Supplementary figure. [file jcav14p0989s1.pdf]

Additional file

Additional file 1: Figure S1. The heat map shows the different strengths of genes which are associated with the CXC family and inflammatory factors between early and late stages of HNSCC. Gene expression strength is indicated as follows: red, high expression; blue, low expression.

FIGURE S1

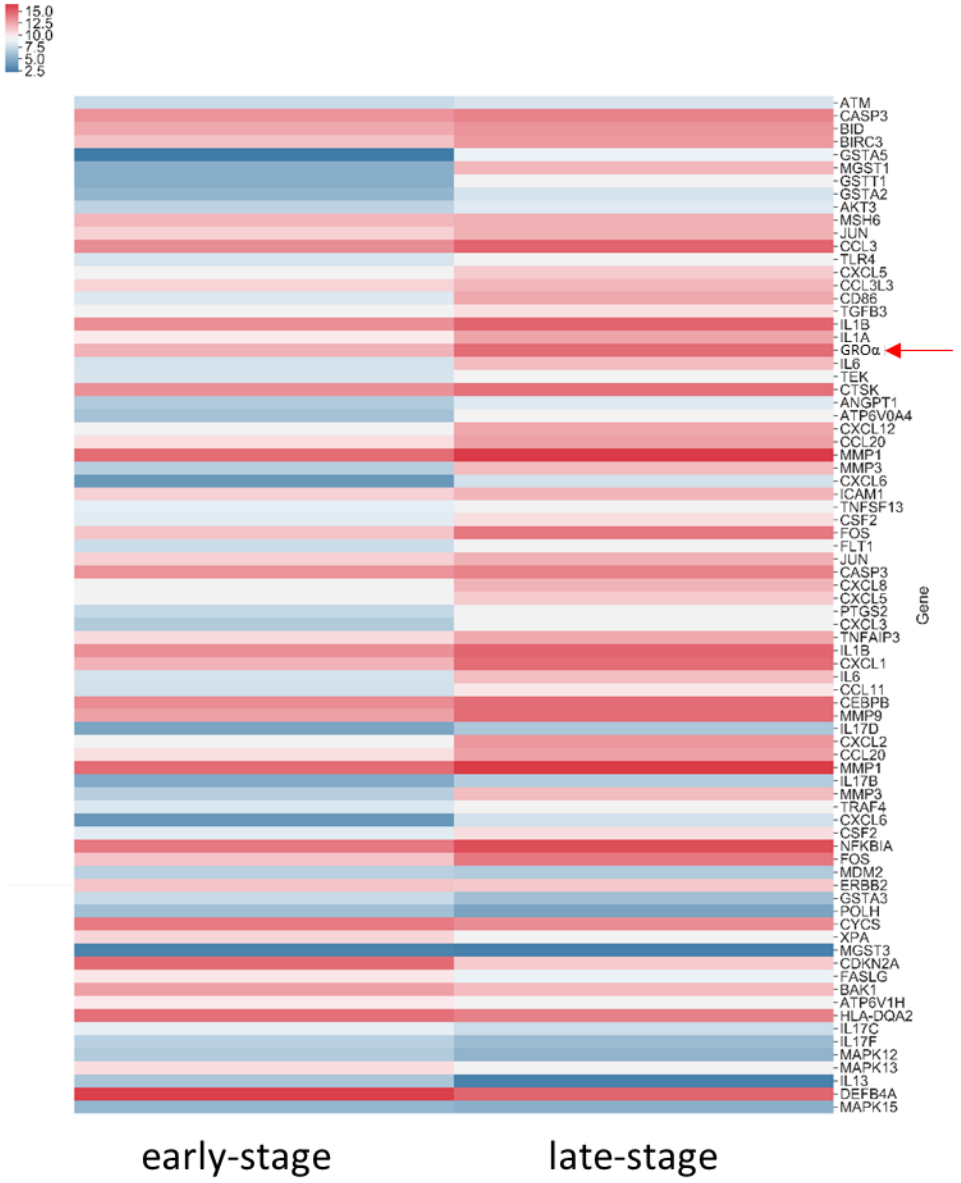
15.0
12.5
10.0
7.5
5.0
2.5
ATM
CASP3
BID
BIRC3
GSTA5
MGST1
GSTT1
GSTA2
AKT3
MSH6
JUN
CCL3
TLR4
CXCL5
CCL3L3
CD86
TGFB3
IL1B
IL1A
GROα
IL6
TEK
CTSK
ANGPT1
ATP6V0A4
CXCL12
CCL20
MMP1
MMP3
CXCL6
ICAM1
TNFSF13
CSF2
FOS
FLT1
JUN
CASP3
CXCL8
CXCL5
PTGS2
CXCL3
TNFAIP3
IL1B
CXCL1
IL6
CCL11
CEBPB
MMP9
IL17D
CXCL2
CCL20
MMP1
IL17B
MMP3
TRAF4
CXCL6
CSF2
NFKBIA
FOS
MDM2
ERBB2
GSTA3
POLH
CYCS
XPA
MGST3
CDKN2A
FASLG
BAK1
ATP6V1H
HLA-DQA2
IL17C
IL17F
MAPK12
MAPK13
IL13
DEFB4A
MAPK15
Gene
early-stage
late-stage
